# Anti-LRP/LR-specific antibody IgG1-iS18 impedes adhesion and invasion of pancreatic cancer and neuroblastoma cells

**DOI:** 10.1186/s12885-016-2953-2

**Published:** 2016-11-24

**Authors:** Thalia M. Rebelo, Carryn J. Chetty, Eloise Ferreira, Stefan F. T. Weiss

**Affiliations:** School of Molecular and Cell Biology, University of the Witwatersrand, Johannesburg, Republic of South Africa (RSA)

**Keywords:** Metastasis, 37 kDa/67-kDa laminin receptor (LRP/LR), Adhesion, Invasion, Laminin-1, Metastasis, Pancreatic cancer, Neuroblastoma

## Abstract

**Background:**

Cancer has become a global burden due to its high incidence and mortality rates, with an estimated 14.1 million cancer cases reported worldwide in 2012 particularly as a result of metastasis. Metastasis involves two crucial steps: adhesion and invasion, and the non-integrin receptor; the 37-kDa/67-kDa laminin receptor precursor/ high affinity laminin receptor (LRP/LR) has been shown to be overexpressed on the surface of tumorigenic cells, thus being implicated in the enhancement of these two crucial steps. The current study investigated the role of LRP/LR on the aggressiveness of pancreatic cancer (AsPC-1) and neuroblastoma (IMR-32) cells with respect to their adhesive and invasive potential.

**Methods:**

AsPC-1 and IMR-32 cells were utilized as the experimental cell lines for the study. Cell surface LRP/LR levels were visualised and quantified on the experimental and control (MCF-7) cell lines via confocal microscopy and flow cytometry, respectively. Total LRP/LR levels in the cell lines were assessed by Western blotting and the adhesive and invasive potential of the above-mentioned cell lines was determined before and after supplementation with the anti-LRP/LR specific antibody IgG1-iS18. Statistical significance of the data was confirmed via the use of the two-tailed student’s *t*-test and Pearson’s correlation coefficient.

**Results:**

Flow cytometry revealed that AsPC-1 and IMR-32 cells displayed significantly higher cell surface LRP/LR levels in comparison to the MCF-7 control cell line. However, Western blotting and subsequent densitometric analysis revealed that all three tumorigenic cell lines displayed no significant difference in total LRP/LR levels. The treatment of AsPC-1 and IMR-32 cells with IgG1-iS18 caused a significant reduction in the adhesive and invasive potential of the cells to laminin-1 and through the ECM-like Matrigel™, respectively. Pearson’s correlation coefficients indicated a high correlation, thus suggesting a directly proportional relationship between cell surface LRP/LR levels and the adhesive and invasive potential of AsPC-1 and IMR-32 cells.

**Conclusion:**

These findings suggest that through the interference of the LRP/LR-laminin-1 interaction, the anti-LRP/LR specific antibody IgG1-iS18 may act as an alternative therapeutic tool for the treatment of metastatic pancreatic cancer and neuroblastoma.

## Background

Cancer is defined as the uncontrolled proliferation of body cells due to the diminished levels of apoptosis or programmed cell death, thus resulting in abnormal cellular growth [1, 2]. Cancer is implicated as the primary cause of death in economically developed countries and the second leading cause of death in developing countries [3]. According to the World Cancer Research Fund (WCRF), 14.1 million cancer cases were reported worldwide in 2012 and this number is expected to increase to 24 million by the year 2035 [4]. Referring to the present study, two cancer types are of importance namely pancreatic cancer which is an aggressive cancer type as it is asymptomatic in the early stages and upon diagnoses the tumour is of an advanced stage, after metastasis has occurred. Pancreatic cancer was ranked as the 12^th^ most diagnosed cancer in 2012 with approximately 338 000 cases being diagnosed. Neuroblastoma is an aggressive and malignant cancer type with 50% of all cases being classified as high risk upon diagnosis as the disease has metastasised. Neuroblastoma most commonly affects infants and young children and it was ranked as the 17^th^ most diagnosed cancer types in 2012 with approximately 256 000 cases being diagnosed [4]. Due to these alarming statistics, it is therefore crucial to develop novel therapeutic strategies for the treatment of cancer and in particular metastatic pancreatic cancer and neuroblastoma.

The 37-kDa/67-kDa laminin receptor (LRP/LR) is a 295 amino acid, transmembrane receptor consisting of three domains; the N-terminal intracellular cytosolic domain, the transmembrane domain and the C-terminal extracellular domain which contains binding sites for laminin-1, carbohydrates, elastin, prion proteins and IgG antibodies [5–7]. The 37-kDa LRP is thought to be the precursor of the 67-kDa high-affinity laminin receptor LR, however the exact mechanism by which the precursor forms the receptor is unknown [8]. The receptor is seen to be overexpressed on the surface of tumorigenic cells such as cervical, lung, prostate and colon [9, 10], breast and oesophageal [11] and liver cancer cells [12], thus serving many physiological functions such as signal transduction, cell cycle progression, cell migration, cell-matrix adhesion, cell viability and proliferation [13–15]. LRP/LR is also associated with the process of Aβ- generation in Alzheimer’s disease [16, 17] as well as enhancing telomerase activity which is seen to play a role in disease progression and chemotherapy resistance in some cancers [18]. LRP/LR overexpression directly promotes the adhesion and invasion of tumorigenic cell lines via the process of metastasis. Metastasis is responsible for secondary tumor formation thus accounting for 90% of cancer- associated deaths [11]. LRP/LR has a high binding affinity for the extracellular matrix (ECM) protein; laminin-1 [14]. Laminins make up the majority of the non-collagenous glycoprotein component when localized in the basement membrane [19]. Laminins support the promotion of the invasive phenotype of several tumorigenic cells as they are involved in significant biological activities such as cell attachment, growth and migration [19]. The main event responsible for tumor invasion is the interaction of cancer cells; in particular LRP/LR with laminin-1, therefore inducing proteolytic activity which involves type IV collagenase which hydrolyses type-IV collagen in the basal lamina, resulting in the metastatic spread of the cancer cells to distant tissues in the body [19].

Hindering the LRP/LR-laminin-1 interaction may be considered an essential tool in treating metastatic cancer. This may be achieved by blocking LRP/LR with IgG1-iS18. IgG1-iS18 is the full length version immunoglobulin G1 (IgG1)-iS18 of the single-chain anti-LRP/LR antibody scFv iS18. It is a monoclonal anti-LRP/LR specific antibody which was designed to specifically target the LRP/LR-laminin-1 interaction and it is characteristic of having a high stability and long half-life of up to 21 days in blood. This approach has therefore been shown to significantly reduce the adhesive and invasive potential of several tumorigenic cells [10–12].

In this study, we investigated whether the anti-LRP/LR specific antibody IgG1-iS18 is capable of impeding the adhesive and invasive potential of pancreatic cancer (AsPC-1) and neuroblastoma (IMR-32) cells.

## Methods

### Cell culture

Poorly invasive, human breast adenocarcinoma (MCF-7) cells, human pancreatic adenocarcinoma (AsPC-1) cells and human neuroblastoma (IMR-32) cells were cultivated in Dulbecco’s Modified Eagle Medium (DMEM), RPMI 1640 and Minimum Essential Media (MEM), respectively. Each cell culture medium was supplemented with 10% FCS and 1% penicillin/ streptomycin, with additional amino acids, vitamins and salts being added as required for complete growth media. All cell lines were cultivated at 37 °C and 5% CO_2._

Cells were seeded and sub-cultured at appropriate dilutions. The cultured cells were washed and detached using phosphate buffered saline (PBS) and 1X Trypsin/Versene, respectively.

### Reagents and antibodies

Laminin-1 (10 μg/ml) obtained from BD Biosciences was used for cell adhesion assays.

Matrigel™ matrix used in cell invasion assays was extracted from the Engelbreth-Holm-Swarm (EHS) mouse sarcoma a tumor rich in extracellular matrix proteins. Once isolated, the Matrigel™ constitutes of approximately 60% laminin, 30% collagen IV, 8% entactin and several growth factors. It was obtained from Corning Inc.

Chloramphenicol acetyltransferase (CAT) antibody was used as the negative control and it was obtained from Sigma-Aldrich.

IgG1-iS18 was used as the treatment antibody (positive control) and it was recombinantly produced in a mammalian expression system as described by Zuber et al., (2008).

### Confocal microscopy

In order to qualitatively visualize the localization of LRP/LR on the cell surface, confocal microscopy was employed. The cells were first seeded onto coverslips and allowed to reach an approximate confluency of 75% before being fixed for 15 min in 4% paraformaldehyde (PFA). This was followed by five PBS washes and the addition of the primary antibody IgG1-iS18 (1:100) diluted in 0.5% BSA. Post an overnight incubation at 4 °C, the coverslips were rinsed thrice in PBS/BSA. After the addition of the goat anti-human IgG FITC- coupled secondary antibody (abcam) that had been diluted in 0.5% BSA, a further 1 h incubation at 4 °C was allowed. Followed by three washes as before, Hoechst stain diluted in PBS was added and incubated for 10 min to allow for the staining of the nucleus. The cells were washed once with PBS and mounted onto a clean slide using GelMount (Sigma-Aldrich). A period of 2 h was allocated for setting to take place and the slides were then stored at 4 °C until visualisation. Importantly, controls included cells treated with the anti-CAT antibody (primary antibody) together with the FITC-coupled secondary antibody and cells treated with the secondary antibody only.

### Flow cytometry- FACS™ analysis

In order to quantitatively determine cell surface LRP/LR levels, flow cytometry was employed. Trypsin/Versene (1X) was used to detach adherent cells, which were then centrifuged at 150 x g for 10 min. Cells were re-suspended and fixed in 4% PFA at 4 °C for 10 min. PFA was discarded and cells were re-suspended in PBS which allowed for the preparation of three cell suspensions, one to which the primary anti-LRP/LR specific IgG1-iS18 antibody was added, one to which anti-CAT antibody was added and one to which no antibody was added (serving as the unstained control). All suspensions were incubated in the dark for 1 h at room temperature. Following three PBS washes by centrifugation at 2700 x g for 5 min, the goat anti-human phycoerythrin (PE)-coupled secondary antibody (abcam) was added to each cell suspension and further incubated for 1 h at room temperature. After the 1 h incubation, suspensions were centrifuged at 2700 x g for 1 min and pellets washed three times in PBS as previously described. The cell suspensions were then analysed using the BD Accuri flow cytometer and software. The experiment was performed in triplicate.

### SDS PAGE and Western blotting

Total LRP/LR levels were determined using sodium dodecyl sulphate polyacrylamide gel electrophoresis (SDS-PAGE) as well as Western blotting. To perform SDS- PAGE, 11 μg of total protein was used. Proteins that were separated according to size by SDS-PAGE were identified via Western blotting. The proteins resolved on the polyacrylamide gel were transferred onto the polyvinylidene fluoride (PVDF) membrane using 1X transfer buffer (20% Methanol in 192 mM glycine and 25 mM Tris) for 45 min at 350 mV and a semi-dry transferring apparatus. Blocking buffer (3% BSA in 1X PBS Tween) was then used to block the blotted membrane for 1 h in order to prevent non-specific binding of primary and secondary antibodies. Once blocked, the membrane was probed with anti- LRP/LR specific primary antibody IgG1-iS18 for 1 h. Prior to the incubation of the membrane in the goat anti-human IgG- Horse radish peroxidase (HRP) conjugated secondary antibody, three 1X PBS Tween (0.1% Tween in 1X PBS) washes were performed. A further three PBS Tween washes were performed after incubation in the secondary antibody, followed by the detection of HRP using an enhanced chemiluminescent Clarity™ Western ECL Blotting Substrate (BIORAD). The resulting fluorescence was detected and visualised using the Chemidoc apparatus (BIORAD). The experiment was executed in triplicate and quantification was performed via densitometric analysis using ImageJ™ software.

### Adhesion assay

In order to analyse the adhesive potential of the tumorigenic cell lines to the basement membrane in vitro*,* laminin-1 (10 μg/ml) was used to coat 96- microwell plates, leaving uncoated wells to be used as negative controls. After coating of the wells for 1 h and washing with 1% BSA in the respective media, other protein binding sites on the well were blocked using 100 μl of 0.5% BSA for 1 h. Cells were trypsinised and diluted in serum-free culture media to a density of 4x10^5^cells/ml and added to the wells in order to assess the adhesive potential. Furthermore, the cells pre-incubated with IgG1-iS18 (0.2 mg/ml) and the anti-CAT antibody (0.2 mg/ml) as the negative control were added to the relevant wells in order to examine the effect the antibody might have on the adhesive potential of the cells. The plates were incubated at 37 °C for 1 h and thereafter the non-adherent cells were washed off with PBS and the adherent cells fixed with 4% PFA for 10 min. The adherent cells were stained with 0.1% crystal violet for 10 min. The stain was extracted using 2% SDS and the absorbance of the extracted dye at 550 nm was assayed as a measure of the adhesive potential using an ELISA reader. The experiments were performed in triplicate.

### Invasion assay

In vitro analysis of the ability of the tumorigenic cell lines to invade the basement membrane in the absence of the anti-LRP/LR specific antibody IgG1-iS18 and when treated with the antibody was assessed using the ECM- like Matrigel™ invasion assay.

Serum-free cold culture medium was used to dilute the Matrigel™ and the diluted gel was dispensed into the upper chamber of a 24 transwell plate (Corning, 8 μm pores). The gel was allowed to solidify for 4 h at 37 °C. After being trypsinised and harvested, the cells were diluted in serum-free culture media at a density of 1x10^6^cells/ml. The cells were then incubated with IgG1-iS18 (0.2 mg/ml) or anti- CAT antibody (0.2 mg/ml) as the negative control and loaded onto the upper-Matrigel™ covered chamber. The lower chamber was then filled with 500 μl of media containing 10% FCS for the test and FCS-free media for the control and incubated for 24 h at 37 °C. After removal of the lower and upper chamber media, the cells were fixed with 100 μl of 4% PFA for 15 min. Cells were then washed with 100 μl cold PBS and further stained using 0.5% toluidine blue dye for 2 min. Non-invasive cells were removed using a cotton swab. The dye was then extracted using 1% SDS and the absorbance measured at 620 nm using an ELISA reader. The experiments were performed in triplicate.

### Statistical evaluation

The two-tailed student’s *t*-test with a confidence interval of 95% was used in order to prove the statistical significance of the results obtained, with *p*-values of less than 0.05 being considered significant. The degree of association between LRP/LR levels and the adhesive/ invasive potential of the cell lines was measured using Pearson’s correlation coefficient. A positive coefficient was an indication of direct proportionality between the two variables; however a negative coefficient implied indirect/ inverse proportionality.

## Results

### Pancreatic cancer and neuroblastoma cells reveal LRP/LR on the cell surface

**Fig. 1:**
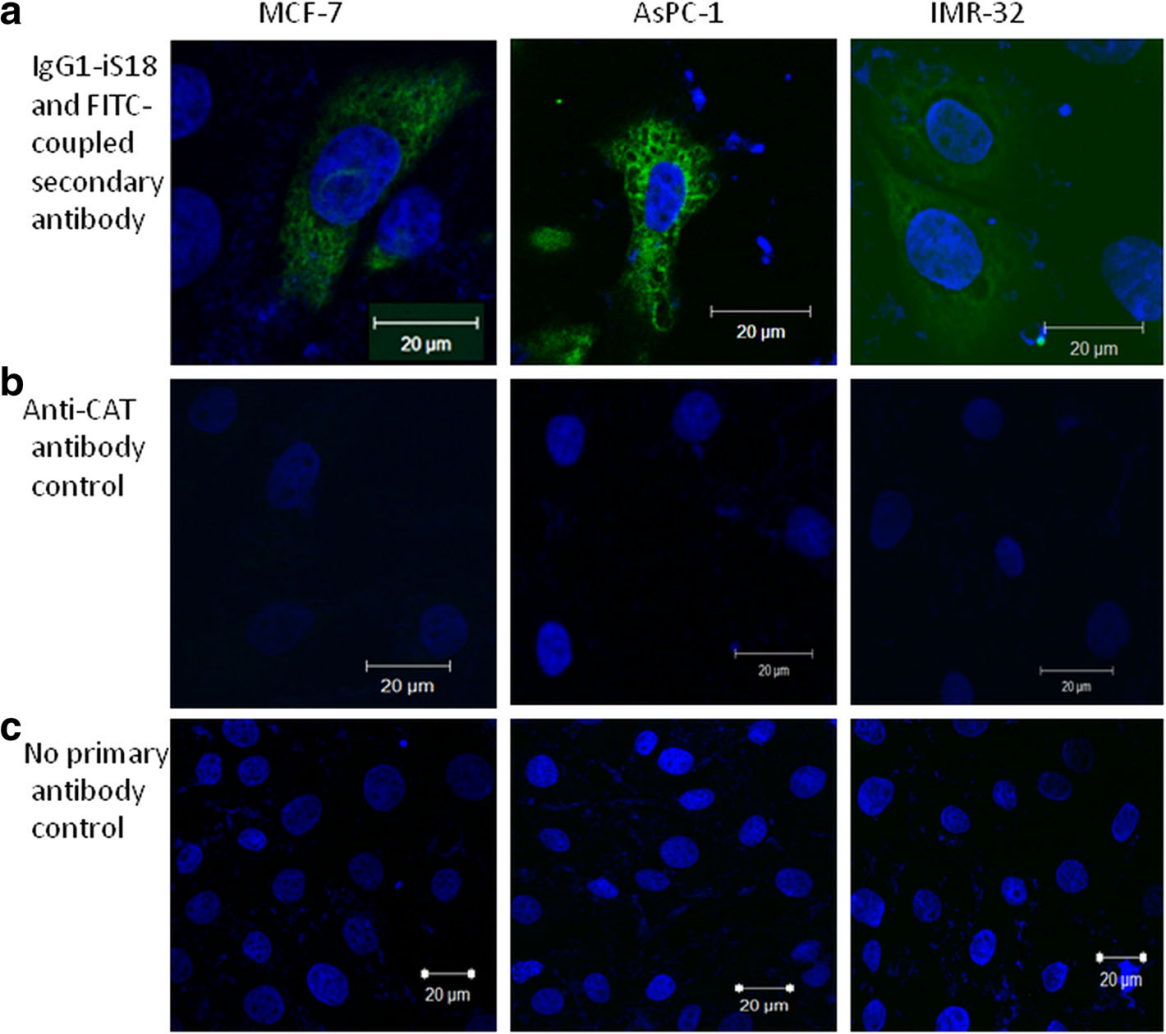
Visualisation of cell surface LRP/LR levels of pancreatic cancer (AsPC-1) and neuroblastoma (IMR-32) cells. The cells were non-permeabilized in order to visualize the surface of the cancerous cells. **a** The cells were labelled with the primary antibody IgG1-iS18 which binds to LRP/LR and the FITC-coupled secondary antibody which is specific for the primary antibody thus exhibiting green fluorescence. **b** The cells were labelled with the anti-chloramphenicol acetyltransferase (CAT) antibody as the negative control. **c** The cells were labelled with the FITC coupled secondary antibody only, to indicate that no non-specific binding of the secondary antibody occurred

Cell surface LRP/LR was visualised in order to confirm that the tumorigenic cells did indeed display LRP/LR on their surface and therefore play a pivotal role in the occurrence of metastasis due to the LRP/LR- laminin-1 interaction. LRP/LR was revealed on the cell surface of the poorly invasive breast cancer control cell line as well as the two experimental cell lines as indicated by the green fluorescence in Fig. 1a. Cells were non- permeablized allowing for cell surface staining of LRP/LR and the secondary antibody was shown to be specific for anti-LRP/LR specific antibody IgG1-iS18 only, as depicted by the control images B) and C) in Fig. 1. The anti-CAT antibody was used as an effective negative control due to its ability to bind specifically to the chloramphenicol acetyltransferase (CAT) bacterial protein which is absent in mammalian cells.

### High percentages of tumorigenic cells reveal LRP/LR on the cell surface

**Fig. 2:**
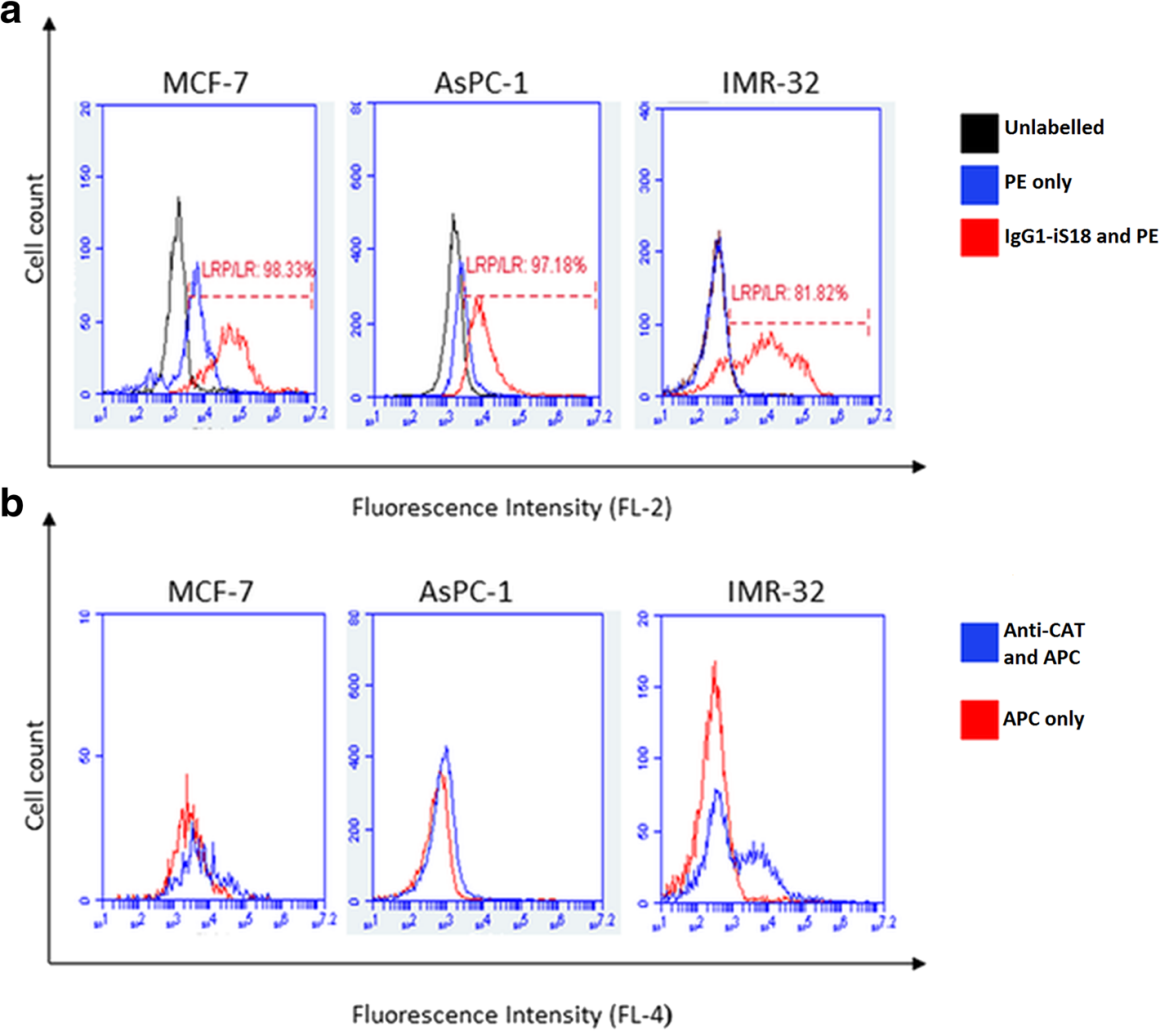
Detection of LRP/LR levels on the surface of pancreatic cancer and neuroblastoma cells. **a** Quantification of LRP/LR on the surface of pancreatic cancer (AsPC-1) and neuroblastoma (IMR-32) cells. The black peak represents the unlabelled cells, whilst the blue peak represents the cells labelled with goat anti-human phycoerythin (PE)-coupled secondary antibody. The red peak represents the cells labelled with both anti-LRP/LR specific antibody IgG1-iS18 and the afore-mentioned secondary antibody. The inclusion of the unlabelled cells as a control, confirms that the secondary antibody does not bind non-specifically. The shift observed between the black and red peak indicates a change in fluorescence intensity thus the presence of LRP/LR on the cell surface of the cell lines under study. **b** Determination of chloramphenicol acetyltransferase protein levels on the surface of the three tumorigenic cell lines as a control. The blue peak represents cells that have been labelled with both anti-CAT primary antibody and goat anti-rabbit allophycocyanin (APC)-coupled secondary antibody, whilst the red peak represents the cells labelled with the afore-mentioned secondary antibody only. No distinct shift is seen between the blue and red peak, thereby indicating the absence of the CAT protein on the surface of the cell lines under study

As confirmed by confocal microscopy, the cells do indeed display LRP/LR on their cell surface, however further quantification of the endogenous expression of cell surface LRP/LR levels was required. Flow cytometry was therefore employed for this quantification. A high percentage of cells within the specific cell population that express LRP/LR on their cell surface were revealed for all three cell lines. This is indicated by the shift between the black (unlabelled) and red (IgG1-iS18 and PE) peaks, as seen in Fig. 2a. Due to the surface staining of the cells with anti-LRP/LR specific antibody IgG1-iS18 and a relevant fluorochrome- conjugated secondary antibody, a change in the fluorescence intensity was observed. Approximately 98.33% of the poorly invasive MCF-7 cells displayed cell surface LRP/LR whilst 97.18% of the AsPC-1 cells and 81.82% of the IMR-32 cells displayed LRP/LR on the cell surface. A shift in fluorescence intensity was not observed in Fig. 2b, thus indicating that the CAT protein was indeed absent on the surface of the three cell lines under study.

### Pancreatic cancer and neuroblastoma cells display significantly higher cell surface LRP/LR levels in comparison to the poorly-invasive breast cancer cells

**Fig. 3:**
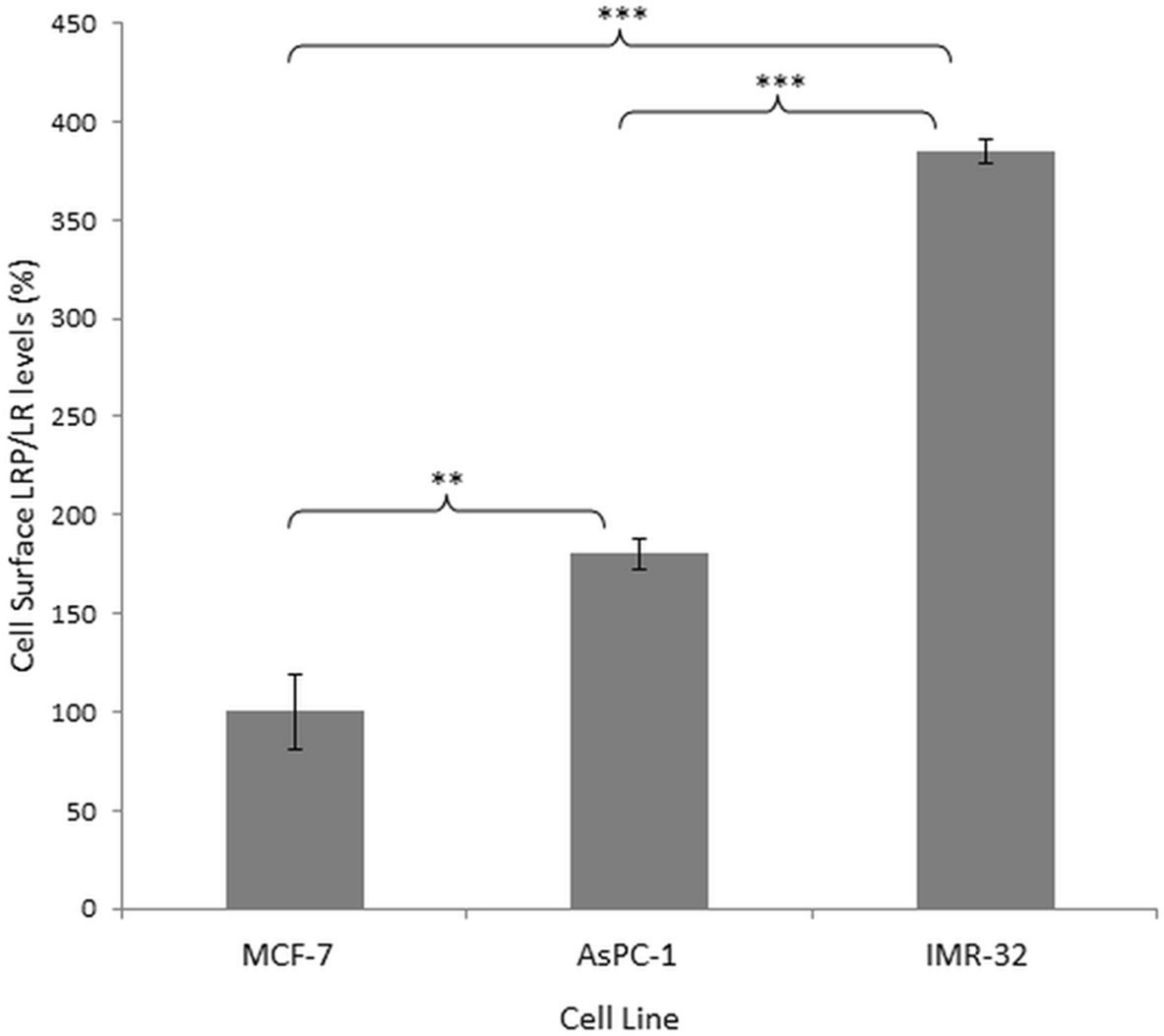
Quantification of cell surface LRP/LR levels on pancreatic cancer (AsPC-1) and neuroblastoma (IMR-32) cells. The cells were labelled with the primary IgG1-iS18 antibody and the anti-human phycoerythrin (PE) secondary antibody. 20 000 cells were analysed across all three cell lines, with the median fluorescence intensity analysed as an indicator of the cell surface LRP/LR levels. In comparison to the MCF-7 control cell line, AsPC-1 cells exhibit an approximate 2-fold increase in cell surface LRP/LR whilst the IMR-32 cells exhibit an approximate 4-fold increase in cell surface LRP/LR. This data is representative of three experimental triplicates. The MCF-7 values were set to 100%. The error bars represent standard deviation and *p*-values *p ≤ 0.05, **p ≤ 0.01, ***p ≤ 0.001

The shift in fluorescence intensity was further analysed using median statistics as a means of determining the amount of cell surface LRP/LR present within the cell population. Identical concentrations of primary (IgG1-iS18) and secondary (PE) antibody were used to label 20 000 cells within a specific cell population over the same time period. The median fluorescence intensity (MFI) is indicative of the differential expression of LRP/LR on the cell surface. A significant difference in surface LRP/LR levels were observed between the poorly invasive MCF-7 cells and the AsPC-1 cells as well as between the IMR-32 cells, as depicted in Fig. 3. When comparing the two experimental cell lines, there was a significant difference in surface LRP/LR levels observed with the IMR-32 cells exhibiting the highest percentage of cell surface LRP/LR.

### Pancreatic cancer and neuroblastoma cells exhibit no significant difference in total LRP/LR levels in comparison to the poorly-invasive breast cancer control cell line

LRP/LR is not only located on the cell surface but it is also found in the nucleus, including the perinuclear region and in the cytosol. Total LRP/LR levels across the two tumorigenic cell lines as well in the poorly invasive MCF-7 cells were determined via Western blot analysis. Further insight on the relationship between the LRP/LR levels, both surface and total and the adhesive and invasive potential of the tumorigenic cells was obtained by performing densitometric analysis.

**Fig. 4:**
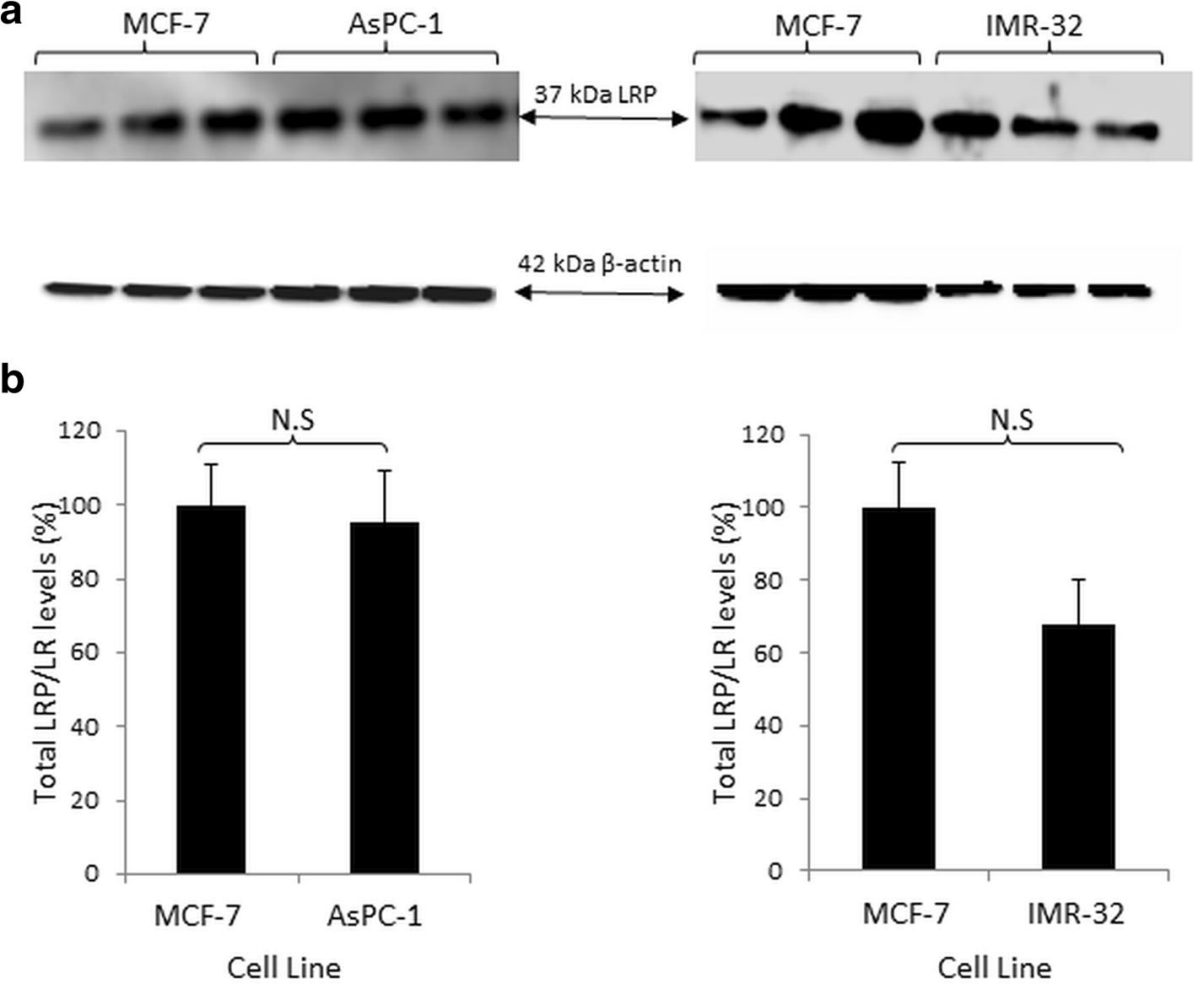
Detection of total LRP levels in pancreatic cancer (AsPC-1) and neuroblastoma (IMR-32) cells. **a** Western blot analysis was performed to detect total 37-kDa LRP levels in all three cancer cell lines. β-actin was used as a loading control. **b** Densitometric quantification performed on these blots revealed that there was no significant difference in total LRP/LR levels between the poorly invasive MCF-7 cells and the two experimental cell lines: AsPC-1 and IMR-32. This data is representative of an average of three experiments. The values obtained from quantification of LRP were divided by the values obtained from β-actin quantification and the resultant values were used to construct the above graph. The MCF-7 values were set to 100%. The error bars represent standard deviation and Non-significant (N.S) *p*-value: *p* > 0.05

As seen in Fig. 4a, LRP/LR was expressed by the three cell lines, but a non- significant difference in total LRP/LR levels were observed (Fig. 4b). The anti-LRP/LR specific antibody IgG1-iS18 only detected the 37-kDa laminin receptor precursor (LRP) form successfully and β-actin served as the loading control.

### IgG1-iS18 significantly reduces the adhesive potential of pancreatic cancer and neuroblastoma cells

**Fig. 5:**
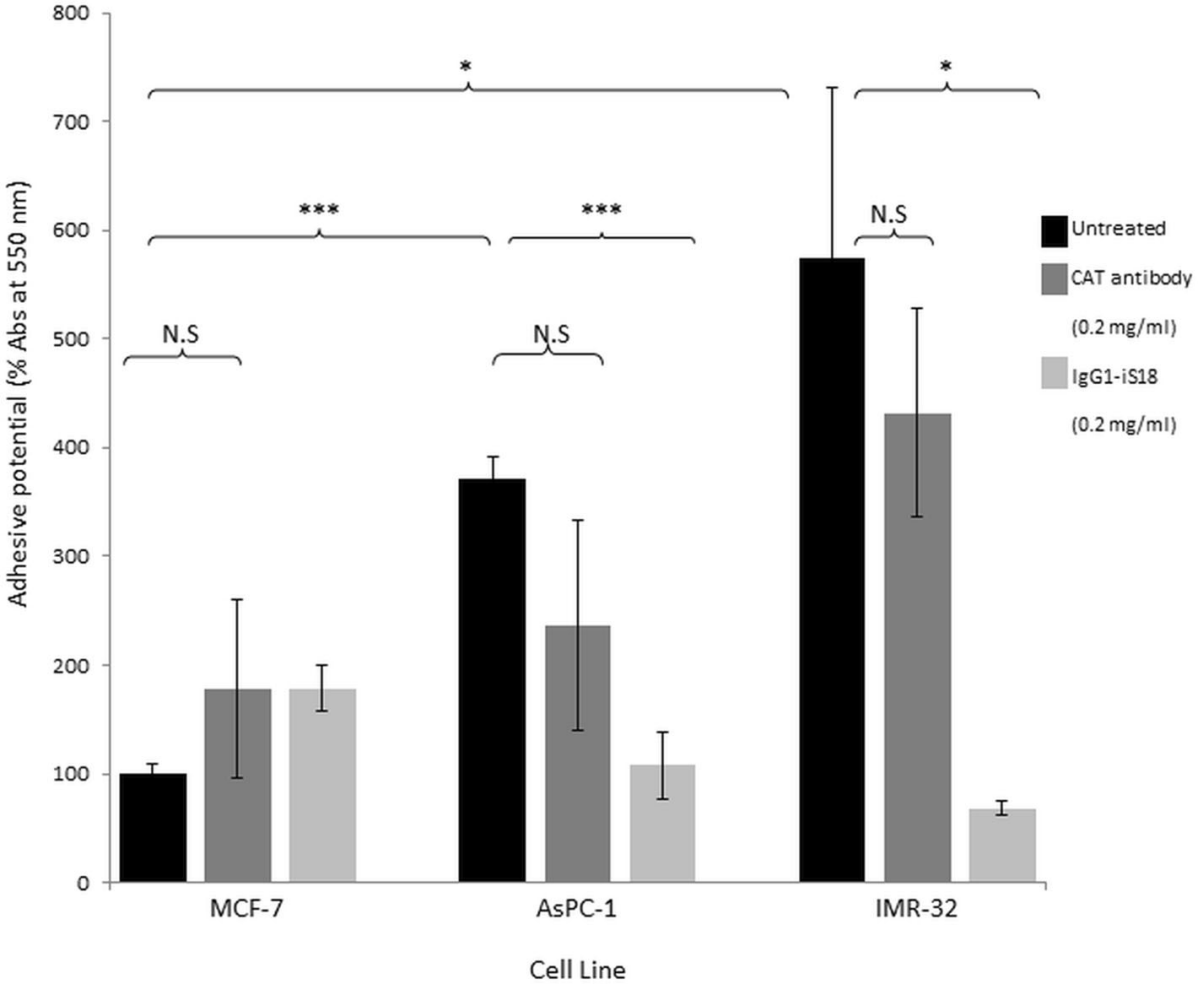
IgG1-iS18 reduces the adhesive potential of AsPC-1 and IMR-32 cells to laminin-1. Absorbance was measured at 550 nm after an incubation of one hour and after staining with crystal violet. CAT was used as a negative control. When treated with IgG1-iS18, the adhesive potential of the AsPC-1 and IMR-32 cells decreased significantly. The MCF-7 untreated value was set to 100%. The error bars represent standard deviation and *p*-values *p ≤ 0.05, **p ≤ 0.01, ***p ≤ 0.001, Non-significant (N.S): *p* > 0.05

The initiation of invasion and ultimately metastasis occurs via the adhesion of a tumorigenic cell to the basement membrane through the LRP/LR- laminin-1 interaction. The LRP/LR-laminin-1 interaction results in the occurrence of additional interactions that enable basement membrane degradation. The tumorigenic cells were treated with IgG1-iS18 and anti-CAT (control) antibodies (0.2 mg/ml) and the absorbance readings taken were a representation of the extent of cell adhesion to laminin-1. The pancreatic cancer cells and the neuroblastoma cells revealed a higher adhesive potential than the poorly- invasive breast cancer (MCF-7) control cell line, with the IMR-32 cells displaying the highest adhesion to laminin-1 as shown in Fig. 5. However, when the cells were treated with the IgG1-iS18 antibody, there was a significant reduction in the adhesive potential of both AsPC-1 and IMR-32 cell lines. The adhesive potential of the tumorigenic cell lines were not significantly affected by the anti-CAT control antibody, as expected.

### Invasion of pancreatic cancer and neuroblastoma cells is significantly reduced by IgG1-iS18

**Fig. 6:**
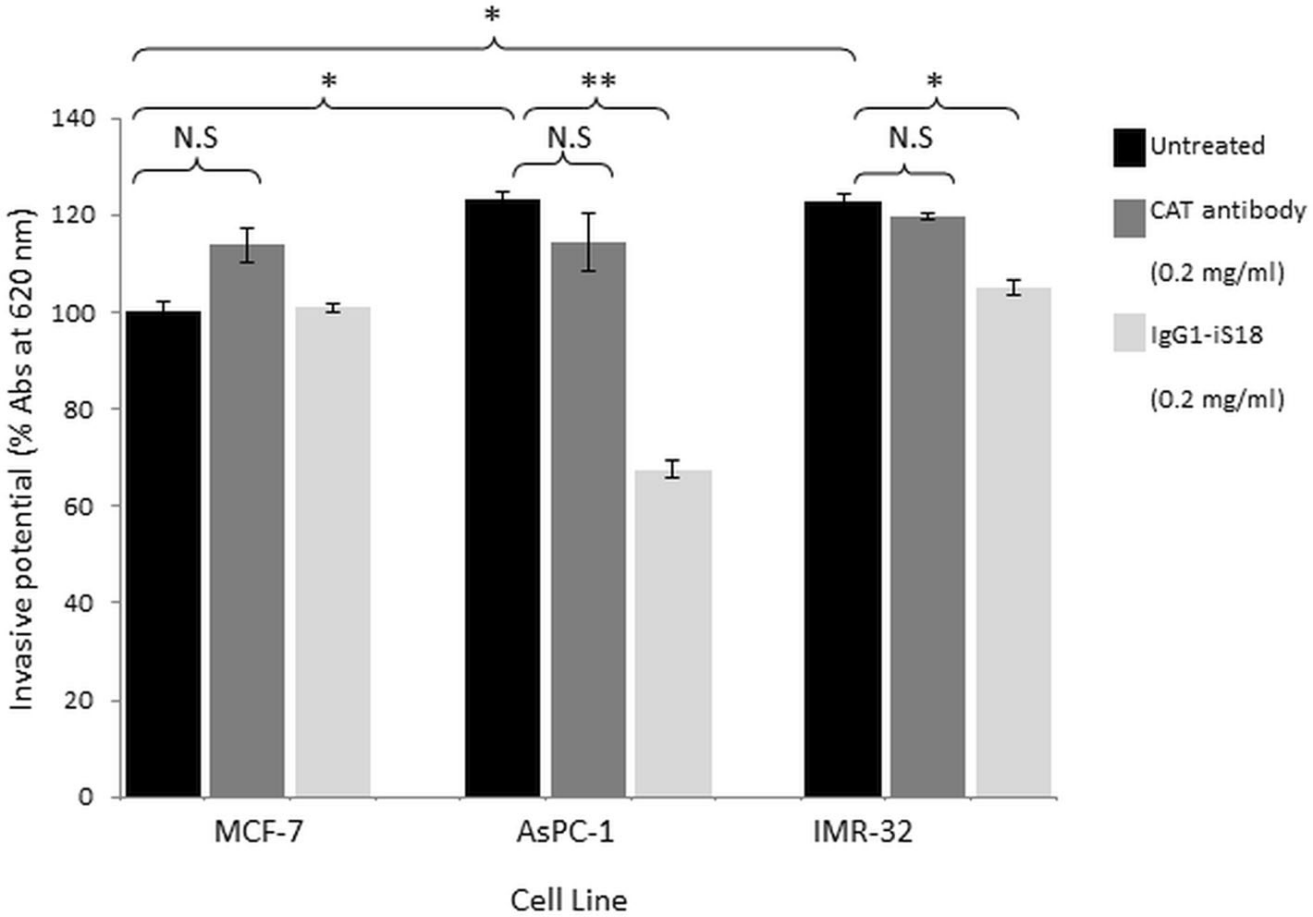
IgG1-iS18 reduces the invasive potential of AsPC-1 and IMR-32 cells through the ECM-like Matrigel™. Absorbance was measured at 620 nm after an incubation of 24 h and after staining with toluidine blue. CAT was used as a negative control. When treated with IgG1-iS18, the invasive potential of the AsPC-1 and IMR-32 cells was significantly decreased, respectively. The MCF-7 untreated value was set to 100%. The error bars represent standard deviation and *p*-values *p ≤ 0.05, **p ≤ 0.01, ***p ≤ 0.001, Non-significant (N.S): *p* > 0.05

Invasion of the basement membrane by the tumorigenic cells is a pre-requisite for the translocation of the cell to secondary sites within the body via the process of metastasis. Matrigel™ invasion assays mimic the components of the basement membrane, allowing for the determination of the invasive potential of the respective cell lines. The three cell lines under study were treated with anti-LRP/LR specific antibody IgG1-iS18 and anti-CAT control antibody (0.2 mg/ml). AsPC-1 and IMR-32 cells proved to be significantly more invasive in comparison to the poorly-invasive MCF-7 control cell line (Fig. 6). Upon treatment with IgG1- iS18, the invasive potential of the pancreatic cancer and neuroblastoma cells were successfully reduced. The invasive potential of the tumorigenic cell lines were not significantly affected by the anti-CAT control antibody, as expected.

## Discussion

Laminin is a key glycoprotein of the basement membrane and it is involved in the attachment, spreading, migration and differentiation of normal and neoplastic cells via the interaction with 37-kDa/67-kDa LRP/LR [20]. The overexpression of the 37-kDa/ 67-kDa LRP/LR in numerous tumorigenic cell lines has been implicated in the enhancement of the LRP/LR-laminin-1 interaction, as indicated by several studies [21, 22]. This enhanced interaction is therefore responsible for invasion of the basement membrane and consequent metastasis [21, 22]. Therefore, inhibiting the LRP/LR-laminin-1 interaction may be effective in impeding metastasis. This has recently been shown, where the anti-LRP/LR specific antibody IgG1-iS18 has significantly reduced the adhesive and invasive potential of cervical, lung, prostate, colon, oesophageal, breast and liver cancer cells [10–12]. In addition, Zuber et al. [23] showed that downregulation of LRP/LR with recombinant lentiviral plasmids expressing anti-LRP siRNAs, caused a reduction in invasive potential of tumorigenic HT1080 fibrosarcoma cells. The present study investigated the role of LRP/LR on the metastatic potential of pancreatic cancer (AsPC-1) and neuroblastoma (IMR-32) cells with respect to adhesion and invasion and whether targeting the LRP/LR-laminin-1 interaction using the anti-LRP/LR specific antibody IgG1-iS18 may impede the metastatic potential of the two cancer cell lines.

All three cell lines displayed LRP/LR on the cell surface as revealed by confocal microscopy. This technique is limited by the fact that it is not quantitative, therefore further analysis using flow cytometry as well as fluorescence intensity was required in order to establish the level at which LRP/LR is displayed on the cell surface of the tumorigenic cell lines. A high percentage of cells with LRP/LR on their cell surface were displayed in the three cell lines under study. In comparison to the control cell line, it was observed that AsPC-1 cancer cells revealed significantly higher and IMR-32 cells revealed the highest cell surface LRP/LR levels. AsPC-1 and IMR-32 cancer cells are classified as metastatic, thus the high percentage of these cells displaying cell surface LRP/LR may be owing to their aggressiveness, with respect to their invasive potential.

All three cancer cell lines did display relatively similar percentages of cells that contain cell surface LRP/LR; however upon additional quantification of cell surface LRP/LR levels by analysis of fluorescence intensity, it was observed that the level of LRP/LR displayed on the cell surface of the invasive cells in comparison to the poorly invasive cells was significantly higher.

Further analysis by Western blotting revealed the expression of the 37-kDa LRP in all three cancer cell lines, but upon densitometric analysis, the poorly invasive and invasive cell lines revealed similar total LRP/LR levels and no significant differences were therefore observed.

Total LRP/LR levels refer to cytosolic and nuclear LRP/LR in the tumorigenic cells, which serves to facilitate translational processes and maintain nuclear structures, respectively. Therefore, although there was no significant difference in total LRP/LR levels between the invasive and poorly invasive cell lines, cell surface LRP/LR levels are of importance for adhesion and consequent invasion of the cancer cell lines under study.

The correlation between cell surface LRP/LR levels to the adhesive and invasive potential of pancreatic cancer (AsPC-1) and neuroblastoma (IMR-32) cells was analysed and a high correlation was detected (Table 1). The respective correlation coefficients indicate a directly proportional and positive relationship between the two factors. This therefore confirms that the enhanced adhesive and invasive potential of the metastatic cancer cell lines under study is as a result of the high cell surface LRP/LR levels [9]. The high correlation coefficients obtained for the adhesive to invasive potential serves as confirmation that the occurrence of adhesion is a pre-requisite for invasion. A non- significant difference in total LRP/LR levels between the cancer cell lines under study was observed and therefore only cell surface LRP/LR levels were taken into consideration in the calculations of Pearson’s correlation coefficients.

**Table 1:** Pearson’s correlation coefficients between cell surface LRP/LR levels and the adhesive and invasive potential of pancreatic cancer (AsPC-1) and neuroblastoma (IMR-32) cells

	LRP/LR levels to adhesive potential	LRP/LR levels to invasive potential	Adhesive to invasive potential
AsPC-1	0.996	0.981	0.994
IMR-32	0.761	0.993	0.833

The variation in the adhesive and invasive potential of both AsPC-1 and IMR-32 cell lines in comparison to the MCF-7 control cell line and how this variation may be due to differences in cell surface LRP/LR levels are demonstrated in the present study. Affirmation that the LRP/LR-laminin-1 interaction facilitates adhesion but also stimulates the secretion of enzymatic substances that degrade the basement membrane such as type IV collagenase (including matrix metalloproteinases, MMPs) is in agreement with reported results, thus suggesting the promotion of invasion and migration through the body [20]. Hence, the higher cell surface LRP/LR levels detected for IMR-32 cells could be responsible for the higher adhesion of the metastatic cell to laminin-1 on the basement membrane. However, the neuroblastoma cells did not exhibit an increased invasive potential as anticipated from the high cell surface LRP/LR levels and adhesive potential. This could possibly be as a result of the expression of tissue inhibitors of metalloproteinases (TIMPs). TIMPs could therefore inhibit the activity of type IV collagenase [24], hence degradation of type IV collagen in the Matrigel is prevented, and thus preventing the invasion of IMR-32 cells in vitro [12]. The AsPC-1 cancer cells displayed high cell surface LRP/LR levels in comparison to the MCF-7 control cell line but lower expression levels than the IMR-32 cells. However, these elevated cell surface LRP/LR levels resulted in the enhanced adhesive and invasive potential of AsPC-1 cells.

It was further observed that the IgG1-iS18 antibody significantly decreased the adhesive potential of both AsPC-1 and IMR-32 cells. The invasive potential of both AsPC-1 and IMR-32 cells were also significantly reduced upon IgG1-iS18 application. The inhibition of adhesion; a mandatory step for invasion of the ECM as indicated by the high Pearson’s correlation coefficients, may be the reason for the significant decrease in invasive potential.

These results suggest that the cleavage of the basal lamina is significantly enhanced by the LRP/LR-laminin-1 interaction, thus facilitating the process of invasion and migration. Furthermore, the exact mechanism by which the anti-LRP/LR specific antibody IgG1-iS18 impedes adhesion and invasion is not fully understood. However, it is possible that the two laminin binding domains (amino acid 160–180 and 205–229) on the 37-kDa/67-kDa laminin receptor LRP/LR may be directly blocked by the antibody or indirectly leading to conformational changes of the laminin binding domains, thereby changing the affinity of the binding domains for laminin.

Different cancer types exhibit different behavioural characteristics; therefore one cannot speculate that the IgG1-iS18 antibody will significantly impede the metastatic potential of all cancer types in the same manner. However, the results of the current study will indicate the use of the antibody as a possible therapeutic tool for the treatment of metastatic pancreatic cancer and neuroblastoma cells. Since LRP/LR is crucial for numerous physiological processes, targeting this receptor may prove difficult as its inhibition may further compromise the health of the patient. Therefore, studies concerning appropriate delivery systems for the IgG1-iS18 antibody should be conducted. Successful animal trials may further illustrate that this antibody is a possible therapeutic tool in the fight against metastatic cancer.

## Conclusion

Anti-LRP/LR specific antibody IgG1-iS18 significantly affected the behaviour of AsPC-1 and IMR-32 cancer cells by impeding their adhesive and invasive potential in vitro, therefore suggesting that this antibody may act as a possible therapeutic tool for the treatment of metastatic pancreatic cancer and neuroblastoma. Future work will involve investigating the efficacy of the IgG1-iS18 antibody as a therapeutic tool for the prevention of several metastatic cancer types, using in vivo mouse xenograft models.

## Abbreviations

APC: Allophycocyanin
AsPC-1: Human pancreatic adenocarcinoma
BCA: Bicinchroninic Acid
BSA: Bovine Serum Albumin
CAT: Chloramphenicol acetyltransferase
DMSO: Dimethyl sulfoxide
ECM: Extracellular Matrix
EDTA: Ethylenediaminetetraacetic acid
FCS: Fetal calf serum
FITC: Fluorescein isothiocyanate
HRP: Horse radish peroxidase
IgG: Immunoglobulin class G
IMR-32: Human neuroblastoma
LR: Laminin Receptor
LRP: Laminin Receptor Precursor
MCF-7: Human breast adenocarcinoma
PAGE: Polyacrylamide gel electrophoresis
PBS: Phosphate Buffered Saline
PE: Phycoerythrin
PFA: Paraformaldehyde
PVDF: Polyvinylidine fluoride
SDS: Sodium Dodecyl Sulfate
